# Selective catalytic hydrogenation of cellulose into sorbitol with Ru-based catalysts

**DOI:** 10.55730/1300-0527.3318

**Published:** 2021-12-06

**Authors:** Ceren ORAK, Aycan SAPMAZ, Aslı YÜKSEL

**Affiliations:** 1 Department of Chemical Engineering, İzmir Institute of Technology, İzmir, Turkey; 2 İzmir Institute of Technology, Geothermal Energy Research and Application Center, İzmir, Turkey

**Keywords:** Hydrothermal liquefaction, Ru-based catalysts, sorbitol, cellulose

## Abstract

Sorbitol is one of the platform chemicals and can be produced from various renewable and sustainable sources via different processes. Hydrothermal liquefaction is an effective and promising approach to produce sorbitol, since the subcritical reaction media and appropriate catalysts provide a selective production of platform chemicals. In this study, sorbitol was produced from different renewable sources (cellulose and glucose) in the presence of Ru-based catalysts (Ru/SiO_2_, Ru/AC, Ru/SBA-15, and Ru/SBA-15-SO_3_) under subcritical conditions. The highest cellulose conversion was achieved as 90% in the presence of Ru/SBA-15-SO_3_ for 1 h of reaction duration. The highest sorbitol yield (%) by hydrothermal liquefaction of cellulose was obtained as 6.2% by using Ru/AC for 1 h of reaction duration. A total of 99.9% of glucose conversion was achieved in the presence of all catalysts. The highest sorbitol yield (%) by hydrothermal liquefaction of glucose was found as 3.8% for 1 h of reaction duration. Owing to the results of GC-MS analysis, the intermediate products were identified, and, thus, a reaction pathway was proposed.

## 1. Introduction

Renewable raw materials, such as biomass wastes, have recently gained great attention in terms of energy and production of building-block or platform chemicals due to the rapid depletion of fossil fuels, the need for green and sustainable energy sources, environmental concerns. The most widely used biomasses as raw materials are cellulose and glucose to produce value-added chemicals in the presence of various catalysts. Cellulose is commonly preferred due to its high chemical stability and insolubility in water [1]. In addition, glucose is the basic sugar unit that could be produced from cellulose via hydrolysis, and more than 50% of its content comprises biomass.

Hydrothermal liquefaction is a promising and efficient approach for the production of various platform chemicals from cellulose using subcritical water (hot-compressed water or pressurized hot water) —its temperature should be between 100 °C and 374 °C under enough pressure to keep its liquid state— since it is a low-cost process which could take place under moderate reaction conditions [2, 3]. Additionally, subcritical water has tuneable properties such as dielectric constant, ion product, viscosity, density, and diffusivity depending on the temperature and pressure. Furthermore, the low viscosity of subcritical water leads to an increase in reaction rates and polarity of water changes concerning the increased temperature, and it becomes nonpolar, so organic compounds could be dissolved [4, 5]. In this technique, firstly, biomass is depolymerized via hydrolysis and decomposed with reactions that take place in subcritical reaction media. After that, owing to dehydration, hydrogenation, deoxygenation, and hydrodeoxygenation reactions, smaller compounds could be obtained [6]. Moreover, the subcritical water environment is very efficient for the conversion of various biomass that supports ionic, polar nonionic, and free radical reactions [7].

Owing to the subcritical water environment and appropriate catalysts, biomass can be converted to selective products with higher conversions under moderate reaction conditions. Furthermore, the usage of catalysts can diminish carbonization and tar formation [8], which can be affected by many factors (i.e., reaction temperature, pressure and time, type of raw material, and catalyst). Acids and bases can act as a catalyst in hydrothermal liquefaction of biomass in the subcritical water environment. For instance, water-soluble products (i.e., sugar, furfural, etc.) can be obtained by the conversion of biomass in the presence of acids [8].

Sorbitol is a natural sugar alcohol and a building-block chemical. Moreover, it is listed as an original platform chemical like organic acids, polyols, etc. Based on the study of Marques et al. 2016, sorbitol is most commonly used in confections and food at a rate of 35% and in toothpaste, toiletries, and cosmetics by 30%. In addition, it could be used in vitamin C synthesis as an intermediate compound, and it comprises 15% of sorbitol usage. In addition to them, sorbitol can also be used in industrial surfactants, pharmaceuticals, and miscellaneous (i.e., polyethers for polyurethanes) [9].

The biotechnological, electrochemical, and chemical methods could be used to produce sorbitol. Among them, the chemical production of sorbitol is the most efficient, most widely used, and a cheap method. Raney nickel (nickel-based) catalysts are commonly used to produce sorbitol from glucose via a hydrogenation reaction. These catalysts have good catalytic activity and are low-cost; however, they show lower selectivity and have a leaching problem into the reaction media, and, thus, the process becomes less economical. In literature, to increase the stability and activity of nickel-based catalysts, Mg, Ti, Fe, Cu, and Mo were incorporated [10]. Furthermore, Mo, Cr, Sn, and Fe were used as promoters to enhance the activity of nickel-based catalysts. However, Mo and Cr containing catalysts were poisoned due to the formation of side products, and Fe and Sn were deactivated in a short reaction time because of the leakage on the surface of the catalyst [11]. The use of catalyst supporting materials (ZrO_2_, SiO_2_, TiO_2,_ and Al_2_O_3,_ etc.) to improve Ni activity and stability has proven to be a good strategy to raise the metal distribution and to increase the surface area. For instance, Geyer et al. studied with ZrO_2_, TiO_2_, ZrO_2_/SiO_2_, ZrO_2_/TiO_2_, and MgO/Al_2_O_3_/SiO_2_ supported Ni for the production of sorbitol, and TiO_2_ content catalysts showed higher catalytic activity compared to other catalysts [12]. Therefore, many researchers have focused their attention on developing other active metal-containing catalysts such as cobalt, platinum, palladium, rhodium, and ruthenium to overcome these disadvantages. Among these supported metal catalysts, only ruthenium (Ru) based catalysts are usable instead of nickel-based catalysts because they show high activity against sorbitol, require less loading, and have less deactivation. Although ruthenium is more expensive than nickel, it is much more active than nickel and less likely to leach [13]. Ruthenium nanoparticles and nano-powders are especially effective catalytic materials for hydrogenation reactions. Hoffer et al. reported that carbon-supported Ru catalysts showed higher selectivity and stability compared to Ni-based catalysts for sorbitol production from sucrose. Additionally, leakage of Ru into the reaction media was not observed [14]. Moreover, carbonized cassava dregs supported Ru nanoparticles catalysts (Ru/CCD), Ru/AC, and Pt/CCD were used to produce sorbitol from glucose [15]. Lazaridis et al. performed a study over the hydrogenation/hydrogenolysis of glucose at low hydrogen pressure (16 bar) and high reaction temperature (453 K) with low glucose concentration (2.7%) using platinum and ruthenium catalysts supported on activated micro/mesoporous carbon (AC) [16]. Deng et al. carried out a study for sorbitol production from cellulose using Ru catalysts loaded on various supports (SiO_2_, CeO_2_, Al_2_O_3_, MgO, and carbon nanotubes (CNT)), and Ru/CNT gave the highest sorbitol yield [17]. Pt/AC-SO_3_H, Pd/AC-SO_3_H, Ru/ AC-SO_3_H, and Ni/AC-SO_3_H were used to produce sorbitol from cellulose, and the higher sorbitol yield was observed with Ru/ AC-SO_3_H [18]. The function of sulfonic acid groups in bifunctional catalysts acts as active sites for acid hydrolysis and hydrogenation of Ru nanoparticles [19]. Therefore, various catalyst support materials (AC, SiO_2_, SBA 15, etc.) were used in different reactions (hydrogenation, photocatalysis, etc.). Among these supporting materials, AC had all the required characteristics, and its textural and surface chemical properties could be tailored so that it became a good catalyst support material. Additionally, it could be stable in acidic and basic media [20]. Moreover, AC has a large surface area and porous structure so that it could adsorb the sample from liquid and gas phase. In addition to them, high surface reactivity and mechanical strength are generally desired properties for ACs. ACs significantly improved the gasification performance of biomass compounds when it was used as catalyst support [21]. SiO_2_, another catalyst supporting material, has high adsorption capacity as well as excellent mechanical properties and a large surface area. Additionally, the silicon hydroxyl groups on their surface exhibit high thermal stability. Furthermore, they are beneficial for the adsorption of compounds [22]. It could be used for several purposes, for instance; Pd-B/SiO_2_ catalyst was used for nitrobenzene hydrogenation, and SiO_2_, as catalyst support material, showed excellent activity during this reaction [23]. SBA 15, which is another catalyst supporting material, has several outstanding properties such as thicker walls, large surface area, and large pores. The dual porosity comprises both mesoporous and intra-wall super-microporous or secondary mesoporous channels, are providing higher stability to the silica framework. Additionally, it could be synthesized using P123, which has low-cost, and it is also biodegradable and nontoxic in nature [24,25]. Consequently, in this study, AC, SiO_2_, SBA15, and SBA15-SO_3_ were chosen as catalyst supporting materials considering their outstanding properties.

Although sorbitol has a large application area with a global market of 3.9 billion by 2020 in the various industrial field, it is not produced in Turkey, so that it was aimed to produce sorbitol from different renewable sources (cellulose and glucose) in the presence of Ru-based catalysts (Ru/SiO_2_, Ru/AC, Ru/SBA-15, and Ru/SBA-15-SO_3_) under subcritical conditions. Although Ru/SiO_2_ and Ru/AC were used to produce sorbitol from cellulose and glucose, it is the first time that Ru/SBA-15 was used to produce sorbitol from glucose, while Ru/SBA-15-SO_3_ was used for sorbitol production from both glucose and cellulose. Additionally, these catalyst supporting materials were widely used for different reactions in literature, and they showed high stability and reusability. Therefore, an experimental study was performed to comprehend their advantages for sorbitol production via hydrogenation of cellulose and glucose. In this context, firstly, a catalyst characterization study (SEM, BET, XRD, and FT-IR) was performed, and then the effect of catalyst supporting materials over catalyst stability at high pressure and temperature was investigated to comprehend the most appropriate catalyst supporting material for this process. Additionally, catalyst amount and reaction duration are crucial parameters for this process, so that their effects were also examined. The proposed reaction mechanism for sorbitol production from glucose was also studied.

## 2. Materials And Methods

### 2.1. Materials

Microcrystalline cellulose and tetraethyl orthosilicate (TEOS) were purchased from Alfa-Aesar. D-Glucose, hydrochloric acid (37%), hydrogen peroxide (H_2_O_2_, 30%), and RuCl_3_•3H_2_O (99.9%) were purchased from Merck. Poly(ethylene glycol)-block (P123), SiO_2_ (nanopowder, 10–20 nm), (3-mercaptopropyl)trimethoxysilane (MPTMS) and activated carbon (powder, 100-mesh particle size) were purchased from Aldrich.

### 2.2. Synthesis of catalysts

#### Ru/SiO_2_

In order to synthesize Ru/SiO_2_, firstly RuCl_3_•3H_2_O (1 g) was dissolved in deionized water (10 mL), and then silica nanoparticles (5%, w/w) were added into Ru-water solution. The obtained solution was dried at 110 °C for 4 h. After completion of the drying step, Ru/SiO_2_ was obtained [26].

#### Ru/AC

Activated carbon (AC) was slowly mixed with an aqueous solution of RuCl_3_•3H_2_O to synthesize 5 w% of Ru containing Ru/AC at room temperature. After that, it was dried at 110 °C for 6 h, and, hence, Ru/AC was obtained [27].

#### Ru/SBA-15

Firstly, solution A containing P123 (2 g) and HCl (70 mL) was prepared. Then, solution B containing TEOS (3.2 mL) and RuCl_3_•3H_2_O in deionized water (5 mL) was prepared based on 0.04 of Ru/Si molar ratio by stirring at room temperature. After that, solution B was added to solution A and stirred at 40°C for 20 h. Then, it was kept in the autoclave at 100 °C for 24 h. Then, it was filtered, washed, and then, the solid residue was dried at 60 °C for 15 h. The solid residue was calcined at 500 °C for 10 h, and Ru/SBA-15 containing approximately 5% Ru was finally obtained [28, 29].

#### Ru/SBA15-SO_3_

Firstly, 4 g of P123 was added into 125 g of HCl (2 M) at room temperature. This mixture was stirred and heated up to 40 °C. Then, the required amount of RuCl_3_•3H_2_O was added into the mixture to obtain 0.1 of Ru/Si molar ratio and stirred for 1 h. After that, the TEOS was added into the reaction media and hydrolyzed for 45 minutes, the MPTMS and aqueous H_2_O_2_ solution were simultaneously added to the solution, and the final mixture was stirred for a further 20 hohurs at 40°C and aged for a further 24 h under static conditions. The solid product was rescued by filtration and then air-dried overnight at room temperature. The P123 template was removed from the synthesized substance by washing ethanol under reflux for 24 h. Finally, the material was washed with ethanol several times and vacuum dried overnight at 60 °C. 1 M of H_2_SO_4_ was mixed with obtained solid residue for sulfonation of Ru/SBA-15. After the filtration, it was dried at 80° C for 12 h. Then, it was calcined at 550 °C for 3 h and finally, Ru/SBA-15-SO_3_ containing approximately 5% Ru was obtained [30,31].

### 2.3. Catalyst characterization

SEM analysis was performed to examine changes in morphological structures of catalysts via the Philips XL 30S FEG device. In this analysis, firstly, catalysts were coated with gold at 24 mA under partial vacuum for 12 min (in the presence of argon) and after coating, they were analyzed by SEM device. The specific surface areas of catalysts were evaluated via BET analysis (device model: Micromeritics ASAP 2010) in a pressure range of 0.1 to 0.25. FT-IR (Shimadzu IR Prestige-21 FTIR 8400S) was used to analyze the bond breakage and new bond formation. The pellets were prepared with 2 mg of each catalyst and KBr to reach a total amount of 150 mg. The analysis was performed with 4 scans in the range of 400–4000 cm^−1^ with a resolution of 4.00 cm^−1^. Low angle X-ray diffraction analysis was performed with Rigaku Ultima-IV by CuKα radiation with a step length of 0.02.

### 2.4. Experimental set-up and procedure

**Figure 1:**
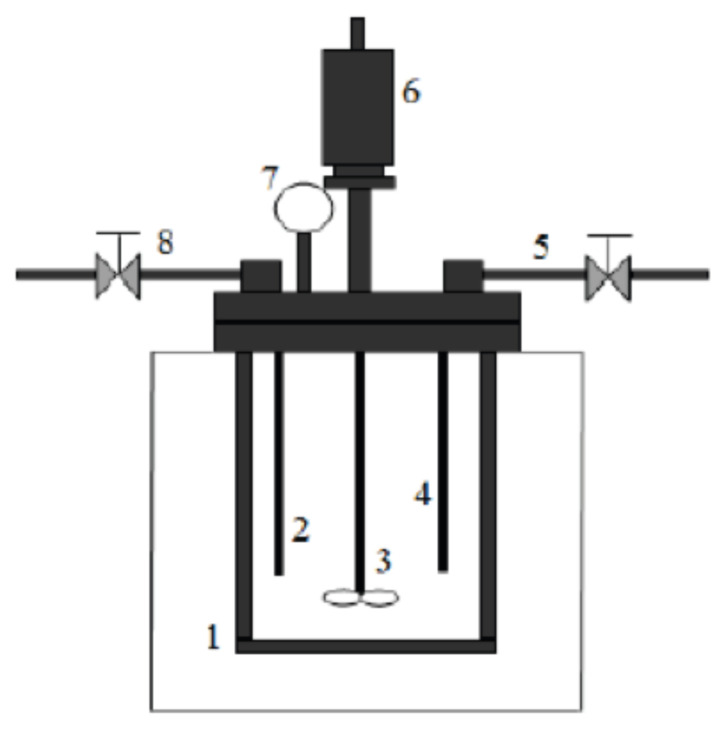
Batch reactor for sorbitol production: 1) stainless steel beaker, 2) thermocouple, 3) stirring impeller, 4) gas inlet, 5) input nitrogen gas, 6) magnetically driven stirrer, 7) pressure gauge, 8) gas sample collecting valve.

The sorbitol production from cellulose and glucose via hydrothermal liquefaction was carried out in a batch reactor (Parr 5500 High-Pressure Compact Reactor, V: 300 mL) made of 316 Stainless Steel and the reactor configuration is illustrated in Figure 1.

**Figure 2:**
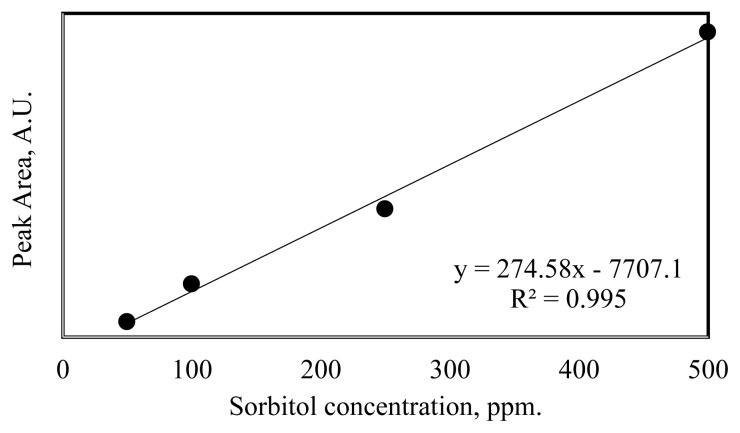
Calibration curve of sorbitol.

The hydrogenation reactions of cellulose and glucose for the production of sorbitol were carried out in the batch reactor by adding 4 g of cellulose and 2.8 g of glucose in 100 mL deionized water using different amounts of synthesized catalysts. After the introduction of raw material and catalyst, the mixture was stirred in the batch reactor and flushed with nitrogen to remove air from the reaction media. After the purging step, the mixture was heated up to 150 °C, and when the system reached the desired temperature, reactions for sorbitol production were carried out (150 °C and 5 bars) for 1- and 2-h reaction time. A chiller was used to keep the reaction temperature constant and to cool it to get samples at the end of the hydrogenation reaction. At the end of the reaction, the system was cooled down to around 50 °C, and the solid and liquid fractions were separated by filter paper. While the liquid samples were analyzed via HPLC, the solid samples were analyzed via FT-IR. HPLC analysis was carried out using refractive index (RI) detector and Shodex Sugar SC1011- 8x300 mm column at 50 °C of column temperature. In this analysis, 5 mM H_2_SO_4_ containing ultra-pure water was used as a mobile phase with a flow rate of 0.5 ml/min. The calibration curve for sorbitol is given in Figure 2.

The conversion of raw material (cellulose and glucose) was calculated by Equation 1 (Eq.1) and the yield of sorbitol was calculated by Equation 2 (Eq. 2).


Eq. 1 $$conversion,\;\%=\frac{m_{0}-m_{1}}{m_{0}}*100$$

Eq. 2 $$yield\;of\;sorbitol,\;\%=\frac{C_{s}}{C_{R}}*100$$

where m_0_: initial mass of raw material (g), m_1_: final mass of raw material (g), C_S_: sorbitol concentration (ppm), C_R_: raw material concentration (ppm).

In order to determine the intermediates of the hydrogenation reaction of glucose, GC-MS (Agilent 6890 N/5973 N Network, USA) analysis was performed. In this analysis, helium (20 ml/min of flow rate) was used as mobile phase, and the GC-TCD detector was used at 250 °C. In addition, the oven temperature program was as follows: 50 °C for 3 min, 100 °C (5 min with an increase of 50 °C/min), 200 °C (5 min with an increase of 50 °C/min), and 250 °C (7 min with an increase of 50 °C/min).

## 3. Results and discussion

### 3.1. Catalyst characterization

**Figure 3:**
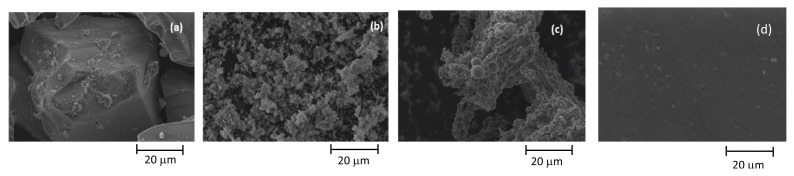
SEM images of synthesized catalysts, (a) Ru/SiO_2_, (b) Ru/SBA-15, (c) Ru/SBA-15-SO_3_, (d) Ru/AC.

SEM, BET, and FT-IR analyses were performed to comprehend the properties of catalysts. SEM diagrams of Ru/SiO_2_, Ru/AC, Ru/SBA-15, and Ru/SBA-15-SO_3_ catalysts are given in Figure 3. It can be stated that these catalysts have a very porous structure. Ru-SBA15 catalyst has uniform dimensions of between 5–6 μm length and 1.3–1.9 μm width on average. Sulfonation of SBA-15 caused an increase in the catalyst activity for hydrolysis compared to SBA-15, and it changed the pore structure of SBA-15. The dimensions of Ru/SBA-15-SO_3_ increased compared to Ru/SBA-15. Additionally, the dimensions of Ru/SiO_2_ were higher than Ru/SBA15-SO_3_ catalyst. Ru/AC showed uniform distribution of Ru particles over activated carbon. BET analysis results of all catalysts were given in Table 1. Additionally, BET areas of pristine SiO_2_, SBA-15, SBA15-SO_3,_ and AC were found as 398, 696, 287, 910 m^2^/g, respectively. The introduction of Ru caused a decrease in the BET areas of all catalysts.

The BET surface area of Ru/SiO_2_ was found as 391.74 m^2^/g, whereas it was reported as 355.3 m^2^/g in literature [32]. Nurunnabi and Turn investigated the pore size effect over the synthesis of Ru/SiO_2_. In this study, four different SiO_2_ which have different BET areas and pore sizes were used. The addition of Ru affected positively the two SiO_2_, which have the following properties: i) surface area: 550 m^2^/g, pore volume: 0.3 mL/g, average pore diameter: 3 nm, (ii) surface area: 450 m^2^/g, pore volume: 0.6 mL/g, average pore diameter: 6 nm. However, the addition of Ru affected adversely the BET area of Ru/SiO_2_, which had a surface area: 300 m^2^/g, pore volume: 1.0 mL/g, average pore diameter: 10 nm [33]. Consequently, it could be deduced that the pore size of SiO_2_ was effective over the BET area of Ru/SiO_2,_ and, hence, the BET area of synthesized catalysts affected positively or negatively depending on their structures. In the present study, the addition of Ru caused an increase in the BET area of Ru/SiO_2_. In the literature, the BET surface area and pore volume of SBA-15 were reported as 725.4 m^2^/g and 4.28 cm^3^/g, respectively [34]. In another study, the BET surface area of the Ru/SBA-15 catalyst was found as 616 m^2^/g [28]. However, the surface area of the synthesized Ru/SBA-15 catalyst was determined to be 527.42 m^2^/g. After the introduction of the sulfone group, the BET surface area of Ru/SBA-15-SO_3_ decreased significantly compared to Ru/SBA-15. Besides, the pore volume and size of Ru/SBA-15-SO_3_ were lower than Ru/SBA-15. For Ru/AC, the BET surface area was 198.97 m^2^/g, while it was reported as 847 m^2^/g in literature [35]. Lazaridis et al. synthesized Ru/AC catalysts containing different amounts of Ru such as 1, 3, and 5%. The BET area of AC was 1175 m^2^/g; however, the BET area of Ru (1 wt.%) /AC was found as 715 m^2^/g [16]. Thus, it could be concluded that the introduction of Ru caused a dramatic decrease in the BET area of the catalyst. In the present study, the BET area of AC decreased drastically with the introduction of Ru.

**Figure 4:**
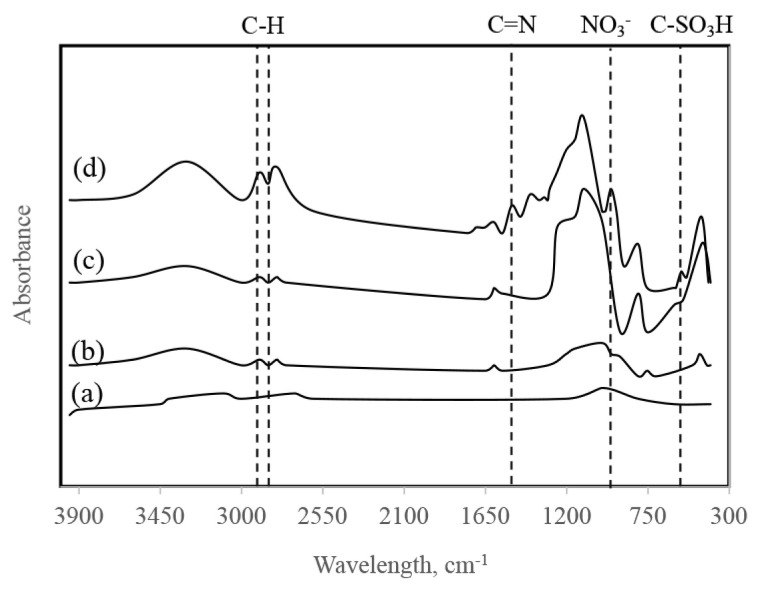
FT-IR spectras of Ru/SBA-15-SO_3_ (a), Ru/SBA-15 (b), Ru/SiO_2_ (c), Ru/AC (d).

FT-IR spectra of the synthesized catalysts are given in Figure 4. In the FT-IR data of Ru/SiO_2_, the bands at 785 cm^−1^ and 459 cm^−1^ attributed to Si-O-Si asymmetrical tensile vibrations, and the broadband around 3431 cm^−1^ refers to the O-H group.

SBA-15 structure has three types of silanol groups (Si-OH). These are germinal, isolated, and hydrogen-bonded, which can act as Brønsted acids, hydrogen bond acceptors, or hydrogen bond donors depending on their range and density [26]. The strong absorbent band at 1105 cm^−1^ in the FT-IR spectrum of Ru/SBA-15 indicates that Si-O-Si asymmetric tensile vibrations in the structure of the catalyst. The bands at around 3478 cm^−1^, 1631 cm^−1,^ and 962 cm^−1^ show the original groups of SBA-15 (Si-OH and Si-O-Si) [35]. The bands at low wavelengths can be associated with metal-oxygen stretching vibrations. For Ru/SBA-15-SO_3_ catalyst, the peak observed at 1000–1200 cm^−1^ represents the S=O band, while the peaks at 939 and 792 cm^−1^ show the presence of Si-O-Si stretching. The weak peak at 1357 cm^−1^ is observed due to the asymmetric stress of the sulfonic acid groups, and, hence, the FT-IR diagram shows that SBA-15 has been successfully functionalized with sulfonic acid groups. Consequently, the presence of characteristic peaks proves that the catalysts were successfully synthesized.

**Figure 5:**
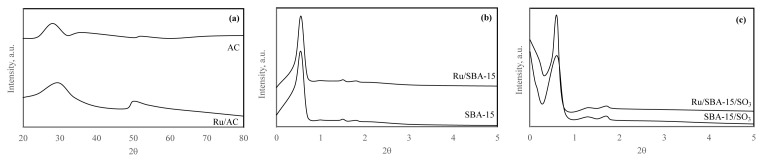
XRD diagrams of AC and Ru/AC (a), SBA-15 and Ru/SBA-15 (b), and SBA-15-SO_3_ and Ru/SBA-15-SO_3_ (c).

XRD diagrams of all supporting materials and catalysts are given in Figure 5. The characteristic peak of AC was slightly shifted after Ru incorporation. The characteristic peaks of SBA15 were observed at certain 2q values that are 0.9 °, 1.7 ° and 1.9 °. Additionally, the peaks at 1.7 ° and 1.9 ° proved that the hexagonal symmetry of SBA-15 was achieved. The intensities of these peaks of Ru/SBA-15 were lower than SBA-15 since the pores of SBA-15 were blocked with Ru particles. However, the hexagonal symmetry was not changed. The characteristic peaks of SBA-15-SO_3_ were observed at certain 2q values that are 0.8°, 1.3° and 2°. A slight shift of peak (2q=0.8°) was noticed because of the introduction of Ru particles, however, the hexagonal symmetry was same [36–39].

### 3.2. Hydrogenation of cellulose and glucose

The hydrogenation of cellulose for the production of sorbitol was carried out at different reaction conditions to understand the effects of supporting material (SiO_2_, AC, SBA-15, and SBA-15-SO_3_), catalyst amount (1 and 2 g), and reaction duration (1 and 2 h). Additionally, the hydrogenation of glucose for the production of sorbitol was carried out at a constant catalyst amount (1.2 g) and different reaction conditions to understand the effects of supporting material (SiO_2_, AC, SBA-15, and SBA-15-SO_3_) and reaction duration (1 and 2 h). The results for cellulose and glucose are given in Table 2 and Table 3, respectively. The conversion of cellulose or glucose and sorbitol yield at 2 h of reaction duration in these tables referred to the time frame between 1 and 2 h of reaction duration. In the absence of any supporting material and/or catalysts, the cellulose conversion was only 4.5%; however, the cellulose conversion enhanced by using catalysts. For instance, the highest total conversion for hydrogenation of cellulose was observed as 92.91% in the presence of Ru/SBA-15-SO_3_ at the end of 2 h of reaction duration, whereas the lowest total conversion was achieved as 14.96% in the presence of Ru/SBA-15 at the end of 2 h of reaction duration. Although the highest conversion was achieved with Ru/SBA-15-SO_3_, the highest sorbitol yield was obtained using Ru/AC. On the other hand, longer reaction durations caused a decrease in the activity of Ru/SBA-15-SO_3_. Due to these reasons, the effect of the catalyst amount was not investigated for 2 h of reaction duration. No sorbitol formation was observed in the presence of Ru/SBA-15 from cellulose via hydrogenation. Besides, the highest conversion was obtained in the presence of 1 g of Ru/SBA-15-SO_3_ for 1 h of reaction duration. Thus, sulfonation of Ru/SBA-15 caused an increase in sorbitol yield due to the acid sites of Ru/SBA-15-SO_3_. However, the highest sorbitol yield was observed in the presence of Ru/AC, which had mesoporous structure for 1 h of reaction duration. Higher conversions were achieved using glucose in the presence of these catalysts, since it was a less stable and degradable compound. In these experiments, 1.2 g of catalysts were used for the hydrogenation of glucose to make a comparison with the literature. The highest sorbitol yield for 1 h of reaction duration was obtained as 3.8% in the presence of Ru/SBA-15-SO_3_. However, this value decreased when the reaction was carried out for 2 h. A similar decrease trend was also observed for hydrogenation cellulose. On the other hand, the highest sorbitol yield for 2 h of reaction duration was obtained as 3.8% in the presence of Ru/SiO_2_. The increase in reaction duration caused an increase in sorbitol yield for Ru/SiO_2_.

### 3.3. Comparison with literature

The results of some related studies in the literature were summarized in Table 4 and stainless-steel autoclave reactors with different volumes were used in all of the given studies. Ru/SBA-15-SO_3_ had higher conversion values (i.e., 90% and 81.8%) for sorbitol production compared to reported values for Ru/SiO_2_ and Ru/AC. Additionally, similar results were observed in this study. For instance, 1.4% of sorbitol yield was achieved using less amount of cellulose and catalyst in a longer reaction duration (10 h), however, in this study, using higher amounts of cellulose and Ru/SiO_2_ in a shorter reaction time (1 h) doubled the sorbitol yield (2.8%). For hydrogenation of cellulose, Ru/SBA-15-SO_3_ showed relatively higher sorbitol yields considering the reaction temperature, pressure, and duration, since relatively lower values of reaction conditions were tested in this study. Hence, the hydrogenation of cellulose could be achieved in milder reaction conditions (i.e., lower reaction duration and temperature) with an alternative catalyst (Ru/SBA-15-SO_3_). Similar conversion values for hydrogenation of glucose were observed in this study and literature. Although higher sorbitol yields were observed for harsh reaction conditions in literature, Ru/SBA-15-SO_3_ could be an alternative for hydrogenation of glucose to sorbitol production at milder reaction conditions in terms of reaction temperature and duration.

### 3.4. Reaction pathway

GC-MS analysis was performed to determine the intermediates, which formed throughout the hydrogenation of glucose in the presence of Ru/SBA-15-SO_3,_ and, thus, a reaction pathway could be developed to comprehend the reaction mechanism. The intermediates and their retention times are listed in Table 5.

**Figure 6:**
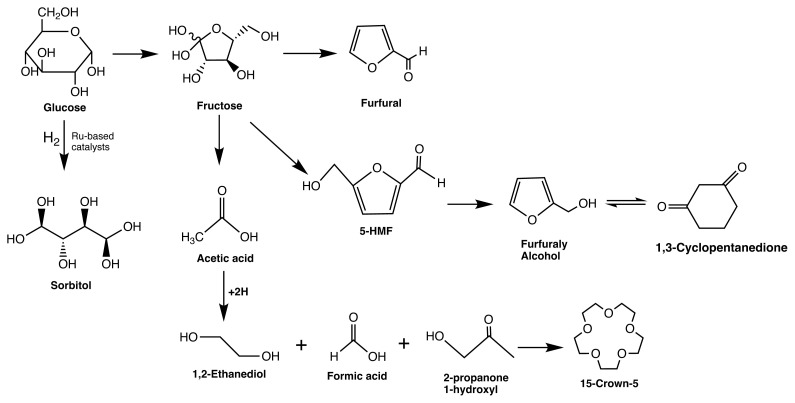
Proposed reaction pathway for sorbitol production via glucose hydrogenation.

Based on GC-MS analysis results, ten different compounds were determined as reaction intermediates. Wang et al. reported that similar intermediates formed during the pyrolysis of cellulose and glucose. In addition, furfural, acetic acid, and 5-hydroxymethyl furfural were detected as intermediate products [41]. Additionally, the proposed reaction pathway is given in Figure 6.

The mechanism of sorbitol formation from glucose includes the dissociative adsorption of molecular hydrogen on hydrogenation metals such as Ru and Ni. The cleavage of the C-O bond and the formation of the hydroxyl group occurs by the following addition of the pair of hydrogen atoms to the hemiacetal group of glucose [16, 42, 43]. It can occur easily on specific metals (Ru, Pt, Ni, etc.) and catalytic systems even at low reaction temperatures and H_2_ pressures of 100–120 °C and 3–8 MPa [36]. The significance of heterogeneous catalysts in this process is that no pH adjustment is required, and, hence, the system could be conserved against corrosion [43]. Therefore, this study showed that Ru-based catalysts could be used for sorbitol production via hydrogenation of glucose. In literature, hydrogenation of cellulose and/or glucose took place for long reaction durations (i.e., 24–48 h) [43]. However, in this study, higher conversion values and yields were achieved in a short reaction time. Consequently, it could be concluded that the Ru-based catalysts, particularly Ru/SBA-15-SO_3_, showed high catalytic activity for sorbitol production via hydrogeneration in short reaction times.

## 4. Conclusion

The building-block chemicals have recently gained great attention to produce from renewable and sustainable sources. Hydrothermal liquefaction is a promising way to produce sorbitol from renewable sources such as cellulose and glucose. Ru-based catalysts (Ru/SiO_2_, Ru/AC, Ru/SBA-15, and Ru/SBA-15-SO_3_) were synthesized to use in hydrothermal liquefaction of cellulose and glucose. A characterization study (SEM, BET, XRD, and FTIR) were carried out, and the results confirmed that the catalysts were successfully synthesized. While the highest cellulose conversion was obtained as 90% using Ru/SBA-15-SO_3_, glucose was almost completely converted in the presence of all Ru-based catalysts. The highest yield% of sorbitol was achieved as 6.2% by hydrothermal liquefaction of cellulose in the presence of Ru/AC catalyst. The intermediate products of hydrogenation of glucose in the presence of Ru/SBA-15-SO_3_ were identified via GC-MS analysis, and, hence, a reaction pathway was proposed based on these results.

## Figures and Tables

**Table 1:** BET areas of catalysts.

Catalysts	BET Surface Area [m^2^/g]	Pore Volume [cm^3^/g]	Pore Size [Å]
Ru/SiO_2_	391.7	0.62	55.24
Ru/SBA-15	527.4	0.56	49.35
Ru/SBA15-SO_3_	168	0.25	47.31
Ru/AC	199	0.16	44.13

**Table 2:** Hydrogenation of Cellulose (Reaction conditions: 4 g of cellulose, 100 mL deionized water, 1–2 g of catalysts, P: 5 bar H_2_, T: 150°C, t: 1–2 h).

Catalyst	Catalyst amount (g)	Time (h)	Conversion (%)	Sorbitol Yield (%)
Ru/AC	1	1	16.4	4.8
Ru/SiO_2_			8.2	0.2
Ru/SBA-15			10.3	-
Ru/SBA-15-SO_3_			90	2.4
Ru/AC	1	2	13.8	5.7
Ru/SiO_2_			10.5	2.7
Ru/SBA-15			5.2	-
Ru/SBA-15-SO_3_			29.1	-
Ru/AC	2	1	25.2	6.2
Ru/SiO_2_			22.5	2.8
Ru/SBA-15			70.4	-
Ru/SBA15-SO_3_			81.8	1.9

**Table 3:** Hydrogenation of glucose (reaction conditions: 2.8 g of glucose, 100 mL deionized water, 1.2 g of catalysts, P: 5 bar H_2_, T: 150°C, t: 1–2 h).

Catalyst	Catalyst amount (g)	Time (h)	Conversion (%)	Sorbitol Yield (%)
Ru/AC	1.2	1	99.9	0.2
Ru/SiO_2_			99.9	2.9
Ru/SBA-15			99.9	-
Ru/SBA-15-SO_3_			99.9	3.8
Ru/AC		2	99.9	0.4
Ru/SiO_2_			99.9	3.8
Ru/SBA-15			99.9	-
Ru/SBA-15-SO_3_			99.9	0.4

**Table 4:** Literature survey for sorbitol production by using Ru-based catalysts.

Catalysts	Reaction Conditions	Conversion,%	Sorbitol Yield,%	Ref.
Ru/SiO_2_	Reactor volume: 50 mL Cellulose amount: 0.25 g Catalyst amount: 0.2 g Water amount: 7.5 mL T: 150°C, P: 4 MPa, t: 10 h	36.2	1.4	19
Ru/SiO_2_	Reactor volume: 100 mL Cellulose amount: 0.05 g Catalyst amount: 0.2 g Water amount: 20 mL T: 185°C, P: 5 MPa, t: 24 h	**-**	**7**	17
Ru/AC	Reactor volume: 1000 mL Cellulose amount: 750 mg Catalyst amount: 300 mg Water amount: 300 mL T: 205°C, P: 5 MPa, t: 1 h	61.1	26.2	40
Ru/AC	Glucose amount: 0.14 g Catalyst amount: 0.06 g Water amount: 5 mL T: 180 °C, P: 1.6 MPa t: 3 h	100	~90 for%3Ru/AC	16

**Table 5:** GC-MS results (Reaction conditions: 2.8 g glucose, 1.2 g of Ru/SBA-15-SO_3_, 100 ml water, 6 bar H_2_, 150 °C, 1 h).

Intermediates	Retention time (min.)
2-Propanone,1-hydroxy-	12.11
Acetic acid	14.56
Furfural	14.94
Formic acid	16.05
1,2-Ethanediol	18.43
Furanmethanol	19.58
1,3-Cyclopentanedione	22.59
2-Propanone, 1-hydroxy	27.40
15-Crown-5	28.55
2-Furancarboxaldehyde, 5- (hydroxymethyl)-	34.68
